# Facile Green, Room-Temperature Synthesis of Gold Nanoparticles Using *Combretum erythrophyllum* Leaf Extract: Antibacterial and Cell Viability Studies against Normal and Cancerous Cells

**DOI:** 10.3390/antibiotics10080893

**Published:** 2021-07-22

**Authors:** Olufunto T. Fanoro, Sundararajan Parani, Rodney Maluleke, Thabang C. Lebepe, Jose R. Varghese, Vuyo Mavumengwana, Oluwatobi S. Oluwafemi

**Affiliations:** 1 Department of Biotechnology, University of Johannesburg, Doornfontein, Johannesburg 2028, South Africa; jolufunto@gmail.com (O.T.F.); vuyom@uj.ac.za (V.M.); 2 Centre for Nanomaterials Sciences Research, University of Johannesburg, Doornfontein, Johannesburg 2028, South Africa; sbarani416@gmail.com (S.P.); rodney.maluleke@gmail.com (R.M.); calvyn.tl@gmail.com (T.C.L.); josv3209@gmail.com (J.R.V.); 3 Department of Chemical Sciences (Formerly Applied Chemistry), University of Johannesburg, Doornfontein, Johannesburg 2028, South Africa

**Keywords:** *Combretum erythrophyllum*, gold nanoparticles, antibacterial, *Staphylococcus aureus*, *Klebsiella pneumoniae*, cytotoxicity

## Abstract

We herein report a facile, green, cost-effective, plant-mediated synthesis of gold nanoparticles (AuNPs) for the first time using *Combretum erythrophyllum* (CE) plant leaves. The synthesis was conducted at room temperature using CE leaf extract serving as a reducing and capping agent. The as-synthesized AuNPs were found to be crystalline, well dispersed, and spherical in shape with an average diameter of 13.20 nm and an excellent stability of over 60 days. The AuNPs showed broad-spectrum antibacterial activities against both pathogenic Gram-positive (*Staphylococcus epidermidis* (ATCC14990), *Staphylococcus aureus* (ATCC 25923), *Mycobacterium smegmatis* (MC 215)) and Gram-negative bacteria (*Proteus mirabilis* (ATCC 7002), *Escherichia coli* (ATCC 25922), *Klebsiella pneumoniae* (ATCC 13822), *Klebsiella oxytoca* (ATCC 8724)), with a minimum inhibition concentration of 62.5 µg/mL. In addition, the as-synthesized AuNPs were highly stable with exceptional cell viability towards normal cells (BHK- 21) and cancerous cancer cell lines (cervical and lung cancer).

## 1. Introduction

Gold nanoparticles (AuNPs) have drawn countless attention [1,2,3] due to their unique properties such as their surface plasmon resonance, wavelength tunability, low-cytotoxicity, competent bioconjugation, enhanced permeability, and retention (EPR) assimilation. These properties offer a prospect for their use in cancer therapeutics, antibiotics, and drug delivery [4,5,6,7]. Several physicochemical methods such as electrochemical techniques, microemulsions, and chemical reduction have been used in the synthesis of AuNPs [8,9]. Although the above-mentioned methods could produce AuNPs with a high yield, they have some limitations, such as high temperatures and the involvement of toxic reducing and stabilizing agents. To circumvent the limitations of these physicochemical methods in the synthesis of AuNPs, studies have been conducted to find simple, cost-effective, benign, and environmentally friendly approaches that involve bacteria [10], fungi [11], biopolymers [12], polysaccharides [13,14], and plant extracts [15,16].

The use of plant extracts in the synthesis of AuNPs is an excellent choice because they are simple, safe, eco-friendly, cost-effective, and biocompatible for biomedical applications [17]. In addition, plant extracts contain different phytochemicals or biomolecules like flavonoids, terpenes, phenols, lectins, alkaloids, tannins, quinones [18], carbohydrates, lipids, reductases proteins, polyphenolic, vitamins [19,20], and among others. Hence, they can serve as both reducing and stabilizing agents, thereby eliminating the use of chemical reagents in the synthesis of AuNPs. These phytochemicals or biomolecules can prevent the agglomeration of nanoparticles(NPs), reduce their toxicity, and increase their bio-assimilation [13,21]. Several plants, seed, flower, and leaf extracts such as *Alcea rosea* [15], *Scutellaria barbata* [22], dragon fruit [23], *Lonicera japonica* [24], and *Salix Alba* [25] have been used for the biosynthesis of AuNPs. 

*Combretum erythrophyllum* (CE) is a plant that is native to South Africa. It grows independently and serves as a shade-providing or an ornamental plant [26]. Different extracts from the leaves, seeds, and bark of CE have been traditionally used for medicinal purposes [27]. Previous studies revealed that its leaves and extracts contain flavonoids, alkaloids, phenolics, and essential oils [28,29], which are excellent biocompatible reducing agents. However, to the best of our knowledge, the use of this versatile plant or its leaf extracts have not been reported for the synthesis of AuNPs. Herein, we report the first synthesis of AuNPs using the aqueous extracts from CE leaves as both reducing and stabilizing agents. The growth of the as-synthesized AuNPs was monitored by using Ultraviolet–Visible (UV-Vis) absorption spectroscopy, while the morphological and structural evaluation was conducted using transmission electron microscopy (TEM) and X-ray diffraction (XRD) analysis, respectively. The biological activity of the as-synthesised AuNPs was evaluated using the minimum inhibition concentration (MIC) for its antibacterial activity against Gram-positive and Gram-negative bacteria. The AuNPs were further evaluated for their cytotoxicity against three cell lines, the normal kidney fibroblast (BHK21), the lunger cancer cell line (A549), and the cervical cancer cell line (Hela). The results revealed that CE-AuNPs exhibited excellent antibacterial activity and biocompatibility.

## 2. Results and Discussions 

### 2.1. UV-Visible Spectra 

**Figure 1:**
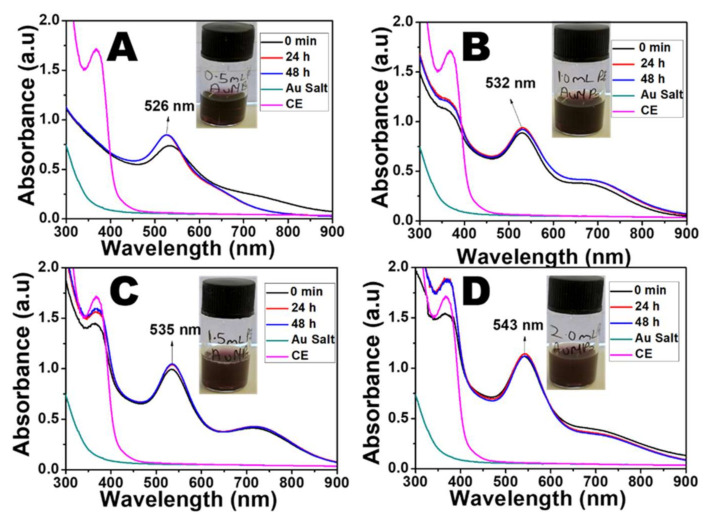
Absorption spectra of CE-AuNPs using (**A**) 0.5 mL CE, (**B**) 1.0 mL CE, (**C**) 1.5 mL CE, and (**D**) 2.0 mL CE.

The green synthesis of the AuNPs was conducted using the extract from dried CE leaves. The colour of the solution changed from golden yellow to light brown upon the addition of the plant extract, indicating the reduction of Au^3+^ ions to Au^0^. The colour intensified as the NPs increased in the reaction medium. The rate of the colour change was dependent on the volume of plant extract added to the Au^3+^ ion solution. As the reaction progressed, a purple colour change was observed at 30 s when 2.0 mL and 1.5 mL CE were used. For the lower volume of CE, it took a little more time to produce the purple colour (40 s for 1.0 mL CE and 2 min 36 s for 0.5 mL CE). This difference in time for the observation of the colour change signifies that the rate of reduction of the Au^3+^ ions is faster with the increase in the extract concentration. The time at which the colour change was observed was taken as 0 min, and further aliquots were collected to monitor the growth of the AuNPs. A distinct surface plasmon resonance (SPR) peak, at 533 nm (0.5 mL CE), 530 nm (1.0 mL CE), 534 nm, (1.5 mL CE), and 541 nm (2.0 mL CE) was recorded for each of the different volumes of CE (Figure 1A–D, Table S1). 

In addition, another shoulder peak was recorded in the near-infrared region (NIR), indicating the possibility of other shapes of AuNPs in this reaction mixture. It was observed that for 0.5 mL CE, there was a blue shift from 533 nm to 526 nm in the SPR peak and the flattening of the shoulder peak after 24 h. This may be due to the size reduction and reconstruction of the AuNPs that were initially formed (Figure S1A,B). However, for the higher CE volumes, the absorption peaks remained in the same position with very little shift in the SPR but showed an increase in absorbance throughout the reaction. As shown in Table S1 and Figure S2A–C, there was a redshift in the SPR peaks of the AuNPs with the increased volume of CE.

Furthermore, an increase in absorbance was observed as the volume of the CE increased, indicating increased in the amount of NPs present in the solution. The peak of the CE was observed at 365 nm, which is significantly different from that of the AuNPs. Additionally, the gold salt had a different absorption spectrum from that of the AuNPs with no SPR peak. The peak of the CE extract was not evident in the spectra of CE-AuNPs when 0.5 mL CE was used (Figure 1A), signifying that all of the extracts were used up during the reduction. However, this peak was observed when higher volumes were used, indicating an excess amount. The AuNPs prepared with 1 mL and 2 mL CE were selected for further characterization. 

The precise reduction mechanism of NPs by biological sources is not fully understood. However, Mukherjee et.al. [30] proposed a plausible mechanism for the bioreduction of AuNPs. They reported that polyphenolic/alcoholic compounds or aldehydes/ketones with protein biomolecules might be responsible for reducing Au salt to AuNPs. Thus, the phenolic compounds found in the CE extract [29] might have been responsible for reducing the Au salt to AuNPs. In addition, flavonoids, saponins, and tannins are known to be critical in the reduction reaction of NPs [31,32]. Similarly, these metabolites, which are also present in the CE, might have contributed to the reduction process. 

### 2.2. XRD Analysis 

**Figure 2:**
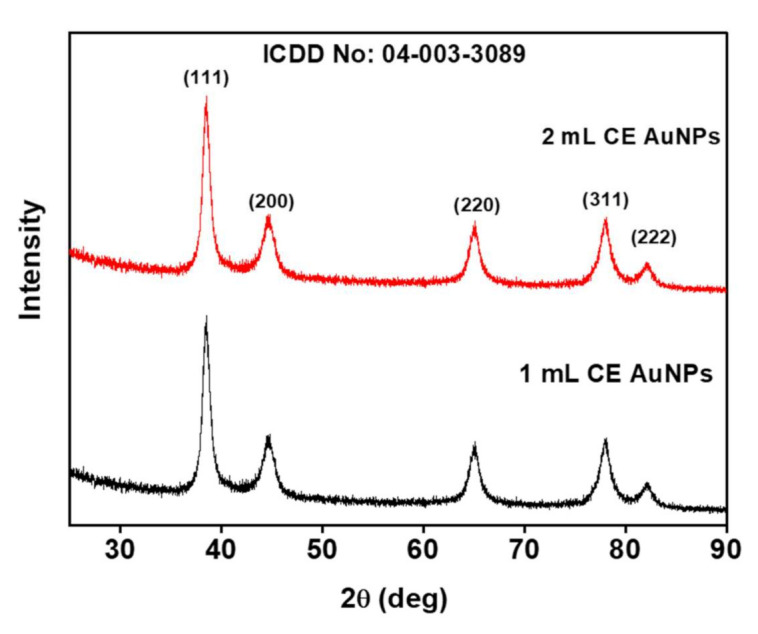
XRD patterns of the as-synthesized CE-AuNPs.

The typical XRD patterns of the CE-AuNPs at 48 h are shown in Figure 2. The diffraction pattern shows peaks at that 2θ values of 38.60°, 48.86°, 65.29°, 78.11°, and 82.34°, corresponding to the (111), (200), 220), (311), and (222) crystallographic planes of the face centred cubic (*fcc*) Au exhibiting the lattice parameter a = 4.08 Å which is in accordance with ICDD no. 04-003-3089. Similar results were reported by Khoshnamvand et.al. [15] in the biosynthesis of AuNPs with *Alcea rosea* leaf extract. The XRD patterns were the same when both the 1 mL CE and 2 mL CE were used in the synthesis. In both cases, the peak corresponding to the (111) plane was more intense, indicating that the nanoparticles were predominantly oriented towards the (111) plane. The width of the (111) peak was used to calculate the average crystallite size of the as-synthesised AuNPs using the Scherrer equation. The calculated average size was 9.26 nm for both the 1 mL and 2 mL CE-AuNPs.

### 2.3. TEM Analysis

**Figure 3:**
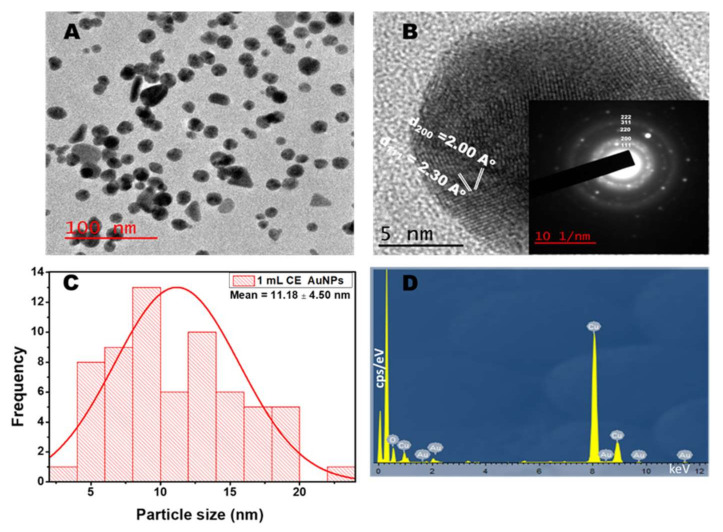
TEM (**A**) and HRTEM (**B**) images of *Combretum erythrophyllum* (CE) synthesised AuNPs inset: SAED (**C**) corresponding particle size distribution and (**D**) EDX spectra of AuNPs using 1 mL CE.

**Figure 4:**
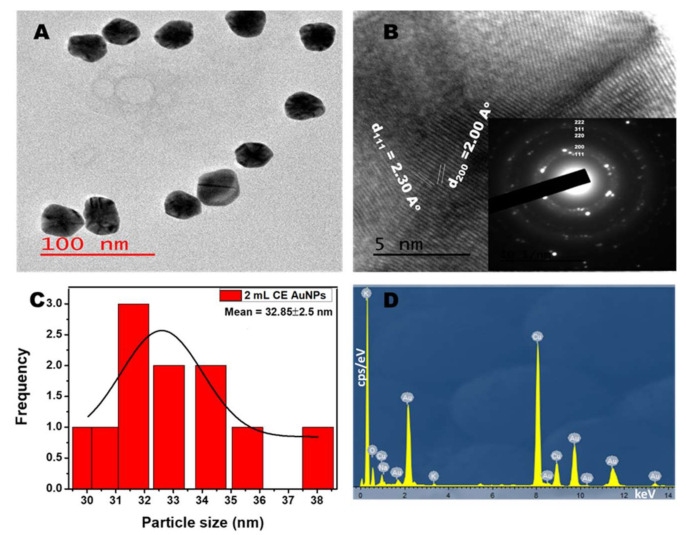
TEM (**A**) and HRTEM (**B**) image of the *Combretum erythrophyllum (CE)* synthesised AuNPs inset: SAED (**C**) corresponding particle size distribution and (**D**) EDX spectra of AuNPs using 2 mL CE.

Figure 3A and Figure 4A show the TEM micrograph of the as-synthesized AuNPs using 1 mL and 2 mL of the CE extract, respectively. The image in Figure 3A shows that the AuNPs prepared using 1 mL CE are polydispersed and mostly spherical. The micrograph also shows a few rod-like and triangle-shaped NPs. The presence of anisotropic shapes corroborates with the SPR peak seen in the NIR (Figure 1B). However, for the synthesis using 2 mL of CE (Figure 4A), the materials are monodispersed, spherical, and are of larger particle sizes. This agrees with its absorption spectra (Figure 1D). The as-synthesized AuNPs showed the presence of lattice fringes, indicating the crystallinity of the AuNPs. The calculated lattice (d) spacing values correlated with the XRD patterns (Figure 3B and Figure 4B). Furthermore, the selected area electron diffraction (SAED) image revealed the ring patterns accompanied by the single spots in a ring (Figure 3B and Figure 4B inset), which agree with the XRD patterns. Figure 3C and Figure 4C show the particle size distribution obtained from the TEM micrograph. The particle size for the 1 mL CE AuNPs ranged between 4.0 nm and 23.0 nm with an average particle diameter of 11.18 ± 4.50 nm, while that of the 2mL AuNPs had an average particle diameter of 32.85 ± 2.50 nm and ranged between 30.0 nm and to 38.0 nm. The narrow size range and higher size recorded in Figure 4C for the 2 mL CE reaction mixture is due to the abundance of CE extract, which is in line with its absorption spectra (Figure 1D) [33]. On the other hand, the 1 mL CE reaction mixture had a long size range and a smaller size, as seen in Figure 3C. A similar size range was reported for the synthesis of AuNPs using *Lonicera japonica* plant extract [24]. The energy dispersive X-ray spectrum (EDX) showed the presence of Au along with other elements such as sodium, oxygen, and potassium, which are present in the plant extract (Figure 3D and Figure 4D). This shows the richness of the CE plant extract in minerals. The observed Cu is due to the copper grid used for the analysis. 

### 2.4. DLS Measurements

The charge and size of NPs significantly determine their effectiveness in biological applications with respect to their stability and distribution in the human body [34]. Figure S3A,B and Table S2 showed that the particle size of the CE-AuNPs NPs was around 26.39 nm and 73.77 nm for 1mL and 2 mL CE-AuNPs, respectively. The size of the as-synthesised NPs is different from the results obtained from the TEM and XRD analyses. This could be attributed to the fact that the DLS evaluates the total hydrodynamic radius of the NPs in solution, which includes conjugated molecules in addition to the particle’s size. Both the CE-AuNPs showed a negative zeta potential of −18.5 mV and −19.2 mV (Figure S3C,D, Table S2) confirming that the NPs have an adequate surface charge for electrostatic and colloidal stability.

### 2.5. Stability of the AuNPs

**Figure 5:**
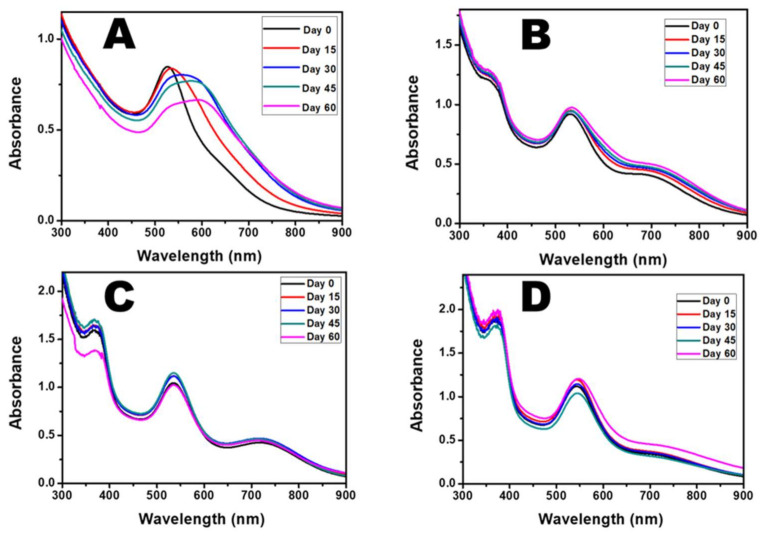
Absorption spectra of as-synthesised-AuNPs after 60 days using (**A**) 0.5 mL CE, (**B**) 1.0 mL CE ,(**C**) 1.5 mL CE, and (**D**) 2.0 mL of CE.

The stability of the NPs is critical to their usefulness in biological applications [30]. The stability of CE-AuNPs was monitored for 60 days using UV-Vis spectroscopy. Figure 5A shows a significant redshift in the wavelength of the AuNPs and a broadening of the plasmon peak as the reaction time increased when 0.5 mL of CE was used. This shows that 0.5 mL of CE extract was only sufficient to reduce the Au salt but not to stabilize it over time. There was no significant shift in the SPR peak for up to 60 days for the rest of the volumes used. These observations indicate that 1.0 mL, 1.5 mL, and 2.0 mL of the CE extract were sufficient to both reduce and stabilize the as-synthesised AuNPs (Figure 5A–D and Table S3).

Furthermore, DLS analysis was performed on the AuNPs synthesised using1 mL and 2 mL of CE over the 60 days. There was no significant difference in the zeta potential of both materials. However, an increase in size was observed for the 1 mL CE AuNPs. This confirms that the 2 mL CE AuNPs will be more stable in biological systems for a longer time compared to the 1 mL and other lower volumes of CE used in the AuNPs synthesis (Figure S4 and Table S4). 

### 2.6. FTIR Analysis

**Figure 6:**
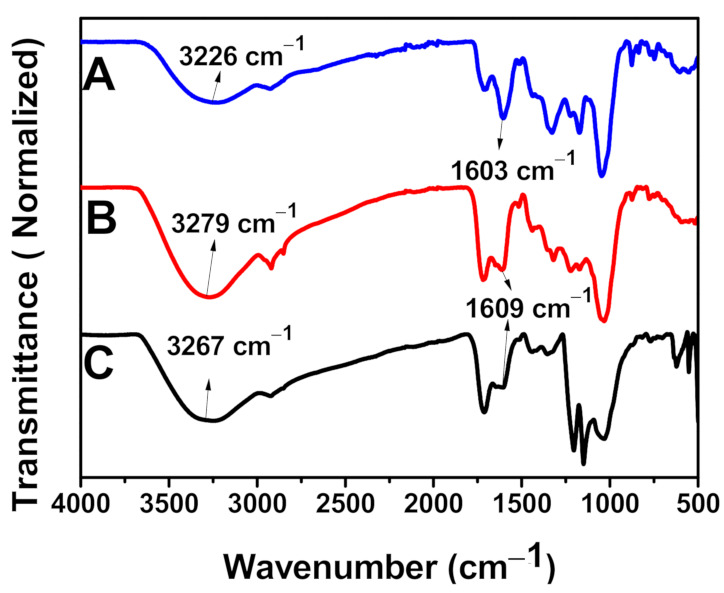
FTIR Spectra of (**A**) CE Extract, (**B**) AuNPs synthesised using 2 mL of CE, and (**C**) AuNPs synthesised using 1 mL of CE.

The functional groups were studied using the FTIR technique to confirm the capping of the AuNPs. FTIR spectra analysis of both the plant extract and NPs showed common band absorptions (Figure 6, Table 1). Specifically, the spectrum of the CE extract show peaks at 3226 cm^−1^, 2923 cm^−1^, 1711 cm^−1^, 1603 cm^−1^,1328 cm^−1^, 1196 cm^−1^, 1035 cm^−1^, 874 cm^−1^, and 747 cm^−1^, which correspond to the O-H stretching of the hydroxyl group found in carbohydrates, polyphenols, etc., the asymmetric stretch of C-H groups, C=O stretching of an ester, the stretching of the carboxylate anion (-COO-), C-N stretching of aromatic amines, S=O stretching, C-O-C stretch, and CH bending, respectively, which comes from polyphenol, carbohydrate, lipids, alkaloids, flavonoids 

The shift observed in the wavenumber of -OH and -COO- stretching of AuNPs compared to the plant extract shows a possible coordination bond between the AuNPs and the plant extract. The addition of metal salts to the plant extract solution at optimal conditions causes the metal ions to swiftly attach to the phytochemicals present in the plant extract through functional groups such as -OH and -COO- and thus become entrapped. This will lead to conformational variation in the biomolecules and exposes the plant’s hydrophobic residues to aqueous phases. This will allow the penetration of reducing agents from the plant extracts and causes the alterations of the entrapped metal into metal nanoparticles [35]. Therefore, it can be suggested that the flavonoids, alkaloids, terpenoids, proteins, and water-soluble biomolecules present in CE served as both reducing and stabilizing agents [29].

## 3. Materials and Method

### 3.1. Materials

Analytical grade chloroauric acid (HAuCl_4_.xH_2_O), 25% glutaraldehyde, 38% formaldehyde, ethanol, Mueller–Hinton agar and broth were procured from Sigma-Aldrich, South Africa. 1 mg/mL streptomycin was purchased from Sigma Aldrich, Switzerland (BCBP5897V). Deionized water was used to prepare all of the aqueous solutions.

### 3.2. Preparation of the Plant Extract

Healthy CE leaves were collected from the Water Sisulu National Botanical Garden at Roodepoort, Johannesburg. The leaves were cleaned followed by drying under ambient conditions. 5 g of the dried leaves were mixed with 100 mL of deionized water and heated at 90 °C for 1 h. The mixture was filtered using a Whatman filter paper, and the filtrate was used for the synthesis.

### 3.3. Green Synthesis of AuNPs

Different volumes of CE Plant leaf extract (0.5 mL, 1.0 mL, 1.5 mL, and 2.0 mL) were each added to 25 mL of 1 mM of HAuCl4 at room temperature followed by stirring at 750 rpm. Aliquots were taken at a specific time interval to monitor the growth of the particles. Each aliquot was purified by centrifugation and washed with deionised water. 

### 3.4. Characterization

The absorption spectra were recorded in the range of 300–900 nm using a Perkin Elmer Lambda 25 UV-Vis spectrophotometer. The shape and size of the as-synthesised AuNPs were determined by transmission electron microscopy (TEM) using a JEOL JEM-2100 at an acceleration voltage of 200 kV. The zeta potential analysis of the AuNPs was studied at 25 °C using a Malvern Panalytical Zetasizer Nano ZS based on the Smoluchowski model. The surface chemistry of the sample was investigated using Perkin Elmer Spectrum Two FTIR spectroscopy over the range of 4000–400 cm^−^^1^. X-ray diffraction (XRD) studies were conducted with monochromatic Cu- Kα1 radiation (λ = 1.54 Å) at the diffraction angle range of 10° and 90° using Bruker D8 Advance X-ray diffractometer.

### 3.5. Antibacterial Activity of AuNPs

The microdilution method was used in the determination of the minimum inhibitory concentration (MIC). Fresh bacterial growths of the listed 18 h cultures of pathogenic bacteria strains *Staphylococcus epidermidis* (Se) (ATCC14990), *Proteus mirabilis* (Pm) (ATCC 7002), *Escherichia coli* (Ec) (ATCC 25922), *Enterobacter cloacae* (Ecl) (ATCC 13047), *Bacillus subtilis* (Bs) (ATCC 19659), *Staphylococcus aureus* (Sa) (ATCC 25923), *Klebsiella pneumoniae* (Kp) (ATCC 13822), *Klebsiella oxytoca* (Ko) (ATCC 8724), *Mycobacterium smegmatis* (Ms) (MC 2155), *Enterococcus faecalis* (Ef) (ATCC 13047), *Proteus vulgaris* (Pv) (ATCC 6380) and *Klebsiella aerogenes* (Ka) (ATCC 27853) were standardized to the 0.5 McFarland standards in Muller–Hilton broth, which was then used as inoculum. In 96-well plates containing 100 µL of 2000, 1000, 500, 250, 125, and 62.5 µg/mL of AuNPs, 100 µL of each inoculum were seeded in triplicate (the starting concentration of AuNPs was determined by a weight to volume ratio whereby 2000 µg of AuNPs were dissolved in 1 mL of deionized water; the subsequent concentrations were obtained by serial dilution). The plates were grown overnight at 37 °C. Muller–Hilton broth (50% *v/v* in DMSO) was used as a negative control. Streptomycin (1 mg/mL) served as the positive control. Viable cells were confirmed in the presence of resazurin dye after 2 h incubation, as they enzymatically reduced resazurin dye (blue color) to the resorufin that fluoresces pink. A pink colour indicated bacterial growth. The smallest concentration that inhibited bacterial growth was recorded as the minimum inhibitory concentration (MIC), and the values were recorded for each bacteria strain.

### 3.6. Morphological Characterization of Bacteria Incubated with AuNPs

Bacterial strains of *S. aureus* (ATCC 25923) and *K. pneumoniae* (ATCC 13822) were each incubated with and without AuNPs (62.5 μg/mL) at 37 °C for 24 h. Samples were collected from both cultures and processed for SEM analysis by adapting the method by [36]. In brief, the samples were centrifuged at 1100× *g* for 10 min, and the supernatant was poured out. This was followed by fixing the pellets at room temperature with (1:1 *v/v*) 1% formaldehyde and 2% glutaraldehyde for 1 h. Subsequently, the samples were centrifuged at 1100× *g* for 10 min, and the supernatant was discarded. Serial dehydration was performed by treating the samples with various ethanol concentrations (30, 50, 70, 90, 95, and 100%) in 10 min intervals. Samples were stored open at 4 °C overnight. The dehydrated samples were mounted on SEM stubs and coated with gold using an emscope SC 500 and viewed with a Tescan VEGA SEM (VEGA 3 LHM, AVG9731276ZA) connected to a monitor at 10 kV. 

### 3.7. Cytotoxicity: MTS Assay 

An MTS (3-(4,5-dimethylthiazol-2-yl)-5-(3-carboxymethoxy-phenyl)-2-(4-sulfophenyl)-2H-tetrazolium) in vitro cytotoxicity assay was conducted to determine the change in cell viability. Briefly, all of the cell lines (normal kidney fibroblasts (BHK21), cancerous cervical HeLa cells, and Lung cancer cells (A549)) were grown using standard tissue culture techniques. The cells (1 × 10^5^ cells/mL) were incubated in 96 well plates at 37 °C overnight with the subsequent addition of the AuNPs in various concentration ranges (3.13–100 μg/mL). The cells were left to incubate for 4 days, whereupon MTS (5 μL) was added to the cells. The absorbance values were measured at 490 nm after 1 h, 2 h, and 4 h incubation periods using a UV-Vis spectroscopy microplate reader. The absorbance was directly proportional to the cell viability. Samples were tested across two plates in triplicate (*n* = 6), and the average value was reported. 

## 4. Biological Studies

### 4.1. Antibacterial Studies

**Figure 7:**
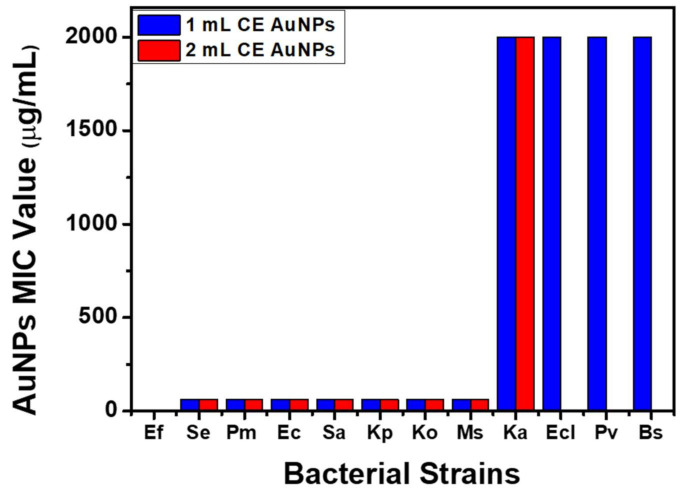
MIC Values of AuNPs using 1 mL and 2 mL of CE.

**Figure 8:**
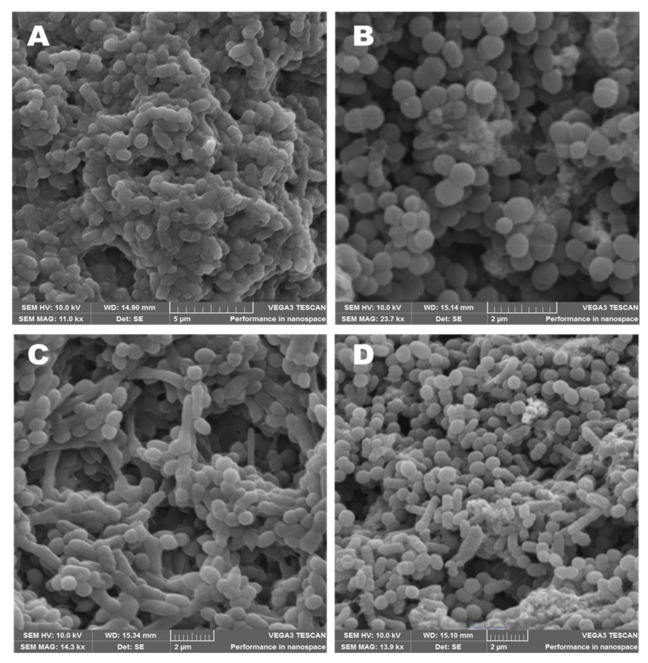
SEM images of (**A**) *S. aureus* after incubation with 62.5 μg/mL of 1 mL CE AuNPs, (**B**) *S. aureus* and (**C**) *K. pneumoniae* after incubation with 62.5 μg/mL of 1 mL CE AuNPs, and (**D**) *K. pneumoniae*.

The as-synthesized AuNPs were evaluated for antibacterial activities against a range of pathogenic Gram-positive (+ve) and Gram-negative (-ve) bacteria listed in Table S5. The as-synthesised AuNPs exhibited broad-spectrum inhibitory activity against both Gram +ve and Gram -ve bacteria (Se, Pm, Ec, Sa, Kp, Ko, and Ms) with a MIC value of 62.5 μg/mL. In addition, the AuNPs synthesised using 1 mL of the extract were potent at a high concentration (2000 μg/mL) against Ka, Ecl, Pv, and Bs. Furthermore, both the 1 mL AuNPs and the 2 mL AuNPs had no inhibitory effect against Ef (Figure 7). A preliminary study evaluating the antibacterial effect of the CE extract showed no effective inhibitory properties compared to the AuNPs (Table S5). SEM analysis was used to study the mechanism of the antibacterial activity of CE-AuNPs against Gram +ve *S. aureus* and Gram -ve *K. pneumoniae.* The nature and degree of cellular membrane damage on the bacteria are seen in bacterial morphology changes (Figure 8A–D). Untreated *S. aureus* had a spherical shape (Figure 8B). After incubation with 1mL AuNPs, S. aureus showed morphological damage and distortion of the cell wall (Figure 8A). This can be attributed to oxidative stress due to the generation of reactive oxygen species causing the detachment of the membrane due to cytoplasm shrinkage. This ultimately leads to a cell wall rupture that culminates in cell death [37,38,39] A similar result was observed with *K. pneumoniae* (Figure 8C). 

### 4.2. Cytotoxicity 

**Figure 9:**
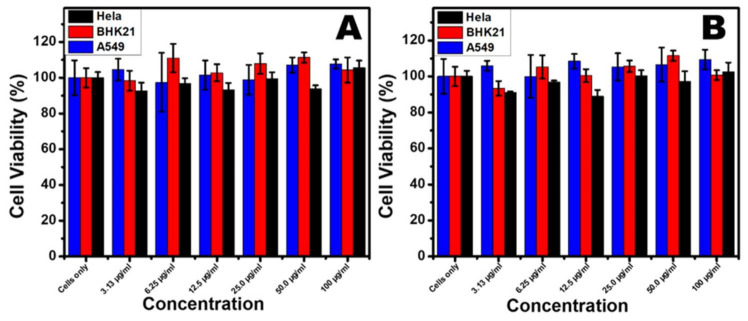
Cell viability results of (**A**) 1 mL CE AuNPs and (**B**) 2 mL CE AuNPs against normal kidney fibroblasts (BHK21), cancerous cervical HeLa cells, and Lung cancer cells (A549).

In this study, cancerous cervical cancer (Hela), lung cancer (A549), and normal kidney fibroblast cells (BHK21) were used to determine the cytotoxicity of CE-AuNPs at different concentrations ranging from (3.13 to 100 μg/mL). Figure 9A,B shows the MTS assay results against these three cell lines. In Figure 9A, the BHK21 cell lines show cell viability ranging from 98% to 111%. This shows that the as-synthesized NPs are highly biocompatible with normal cells at all concentrations. The observed cell viability values above 100% could be attributed to the stimulation of cells by the CE-AuNPs. HeLa cells were also found to be up to 88% viable at a concentration of 12.5 μg/ml. The CE-AuNPs had no significant effect on the lung cancer cell lines. The above similar pattern was observed for all of the cell lines when the AuNPs synthesised with 2 ml-CE were used (Figure 9B). This suggests that the different particle sizes of the as-synthesised AuNPs had no effect on their biocompatibility. Thus, it is noteworthy to mention that the present CE capped AuNPs exhibit excellent cell viability irrespective of the wavelength or size of the AuNPs. Therefore, the results propose that the stabilized AuNPs using *Combretum erythrophyllum* leaf extract can be used for biological applications with both normal and cancerous cells.

## 5. Conclusions

In summary, a facile, cost-effective, green, and eco-friendly process was developed for the synthesis of water-soluble CE stabilized AuNPs. The leaf extract of C. *erythrophyllum* served as the reducing and stabilizing agents. The optical characterisation of the as-synthesised AuNPs via UV-vis and XRD studies revealed the formation of AuNPs with a cubic crystal structure, while FTIR confirmed the capping of the AuNPs by the biomolecules of the CE extract. The study confirmed that the volume of plant extract used for the synthesis determined the SPR wavelength and the size of AuNPs. The CE capped AuNPs are spherical in shape with an average particle size of 13.20 nm and 32.85 nm using 1 mL and 2 mL of CE, respectively. The as-synthesised AuNPs showed broad-spectrum antibacterial activity against both Gram-positive and Gram-negative bacteria. Furthermore, the as-synthesized AuNPs showed high biocompatibility with cancerous and normal cell lines irrespective of the size or wavelength. This makes the as-synthesised AuNPs a viable material for diverse biological applications such as biomedicine, pharmaceutics, cancer therapeutics, and theragnostic. This study provides an excellent starting point for exploring new applications of C. *erythrophyllum* in biomedicine. 

## Figures and Tables

**Table 1:** FTIR Wavenumber and Possible Bonds of CE Extract and its AuNPs.

S/N	CE Extract Wavenumber (cm^−1^)	1.0 mL CE AuNPs Wavenumber (cm^−1^)	2.0 mL CE AuNPs Wavenumber (cm^−1^)	Type of Bond
1	3226	3267	3279	OH- Stretching
2	2923	2929	2915, 2846	C-H Stretching
3	1711	1717	1717	C=O
4	1603.	1609	1609	COO- Stretching
5	1328	1353 1443	1322 1436	C-N Stretching of Aromatic Amine
6	1196	1170 1205	1170 1221	S=O Stretching
7	1035	1042	1042	C-O-C Stretching
8	874	874	881	C-H Bending
9	747	767	767	C-H Bending

## Data Availability

The data presented in this study are available in this manuscript and the supporting document.

## Supplementary Materials

The following are available online at https://www.mdpi.com/article/10.3390/antibiotics10080893/s1, Figure S1: TEM Image of AuNPs synthesized using 0.5 mL of CE (A) at 0 min (B) at 48 h, Figure S2: (A) Absorption spectra of AuNPs using different volumes of CE at 0 min. (B) Absorbance spectra of AuNPs using different volumes of CE at 24 h. (C) Absorbance spectra of AuNPs using different volumes of CE at 48 h, Figure S3: Particle size distribution of AuNPs using (A) 1mL CE and (B) 2 mL CE, Zeta potential distribution of AuNPs using (C) 1 mL CE and (D) 2 mL CE at 48 h, Figure S4: Particle size distribution of AuNPs using (A) 1 mL CE and (B) 2 mL CE, Zeta potential distribution of AuNPs using (C) 1 mL CE and (D) 2 mL CE after 60 days, Table S1: Wavelength and absorption of the AuNPs using different volumes of CE, Table S2: DLS Analysis of AuNPs AT 48 h, Table S3: Wavelength (nm) of AuNPs using different volumes of CE for 60 Days, Table S4: DLS analysis of AuNPs after 60 days, Table S5: Antibacterial activity of AuNPs using 1 mL and 2 mL of CE by MIC.

## References

[1] Shanmugasundaram T., Radhakrishnan M., Gopikrishnan V., Kadirvelu K., Balagurunathan R. (2017). Biocompatible silver, gold and silver/gold alloy nanoparticles for enhanced cancer therapy: In vitro and in vivo perspectives. Nanoscale.

[2] Torrisi L., Torrisi A. (2020). Gold nanoparticles for physics and bio-medicine applications. Radiat. Eff. Defects Solids.

[3] Khan Z.U.H., Khan A., Chen Y., ullah Khan A., Shah N.S., Muhammad N., Murtaza B., Tahir K., Khan F.U., Wan P. (2017). Photo catalytic applications of gold nanoparticles synthesized by green route and electrochemical degradation of phenolic Azo dyes using AuNPs/GC as modified paste electrode. J. Alloys Compd..

[4] Guo Z., Chen Y., Wang Y., Jiang H., Wang X. (2020). Advances and challenges in metallic nanomaterial synthesis and antibacterial applications. J. Mater. Chem. B.

[5] Yoo Y.K., Kim G., Park D., Kim J., Kim Y.S., Yun Kim H., Yang S.H., Lee J.H., Hwang K.S. (2020). Gold nanoparticles assisted sensitivity improvement of interdigitated microelectrodes biosensor for amyloid-β detection in plasma sample. Sensors Actuators B Chem..

[6] Attia Y.A., Farag Y.E., Mohamed Y.M.A., Hussien A.T., Youssef T. (2016). Photo-extracellular synthesis of gold nanoparticles using Baker’s yeast and their anticancer evaluation against Ehrlich ascites carcinoma cells. New J. Chem..

[7] Kang M.S., Lee S.Y., Kim K.S., Han D.W. (2020). State of the art biocompatible gold nanoparticles for cancer theragnosis. Pharmaceutics.

[8] Zou C., Yang B., Bin D., Wang J., Li S., Yang P., Wang C., Shiraishi Y., Du Y. (2017). Electrochemical synthesis of gold nanoparticles decorated flower-like graphene for high sensitivity detection of nitrite. J. Colloid Interface Sci..

[9] Lebepe T.C., Parani S., Oluwafemi O.S. (2020). Graphene oxide-coated gold nanorods: Synthesis and applications. Nanomaterials.

[10] Singh P., Kim Y.J., Zhang D., Yang D.C. (2016). Biological Synthesis of Nanoparticles from Plants and Microorganisms. Trends Biotechnol..

[11] Chowdhury P., Roy B., Mukherjee N., Mukherjee S., Joardar N., Mondal M.K., Roy D., Sinha Babu S.P. (2018). Chitosan biopolymer functionalized gold nanoparticles with controlled cytotoxicity and improved antifilarial efficacy. Adv. Compos. Hybrid Mater..

[12] Devi L., Gupta R., Jain S.K., Singh S., Kesharwani P. (2020). Synthesis, characterization and in vitro assessment of colloidal gold nanoparticles of Gemcitabine with natural polysaccharides for treatment of breast cancer. J. Drug Deliv. Sci. Technol..

[13] Wei S., Wang Y., Tang Z., Hu J., Su R., Lin J., Zhou T., Guo H., Wang N., Xu R. (2020). A size-controlled green synthesis of silver nanoparticles by using the berry extract ofSea Buckthornand their biological activities. New J. Chem..

[14] Amin M., Iram F., Iqbal M.S., Saeed M.Z., Raza M., Alam S. (2013). Arabinoxylan-mediated synthesis of gold and silver nanoparticles having exceptional high stability. Carbohydr. Polym..

[15] Khoshnamvand M., Ashtiani S., Huo C., Saeb S.P., Liu J. (2019). Use of Alcea rosea leaf extract for biomimetic synthesis of gold nanoparticles with innate free radical scavenging and catalytic activities. J. Mol. Struct..

[16] Jose Varghese R., Zikalala N., Sakho E.H.M., Oluwafemi O.S. (2020). Green synthesis protocol on metal oxide nanoparticles using plant extracts. Colloidal Metal Oxide Nanoparticles.

[17] Lakkim V., Reddy M.C., Pallavali R.R., Reddy K.R., Reddy C.V., Inamuddin, Bilgrami A.L., Lomada D. (2020). Green synthesis of silver nanoparticles and evaluation of their antibacterial activity against multidrug-resistant bacteria and wound healing efficacy using a murine model. Antibiotics.

[18] Nazir A. (2020). A review: Use of plant extracts and their phytochemical constituents to control antibiotic resistance in *S. aureus*. Pure Appl. Biol..

[19] A Hussein R., A El-Anssary A. (2019). Plants Secondary Metabolites: The Key Drivers of the Pharmacological Actions of Medicinal Plants. Herbal Medicine.

[20] Nunes M.R., de Souza Maguerroski Castilho M., de Lima Veeck A.P., da Rosa C.G., Noronha C.M., Maciel M.V.O.B., Barreto P.M. (2018). Antioxidant and antimicrobial methylcellulose films containing Lippia alba extract and silver nanoparticles. Carbohydr. Polym..

[21] Santhoshkumar J., Rajeshkumar S., Venkat Kumar S. (2017). Phyto-assisted synthesis, characterization and applications of gold nanoparticles—A review. Biochem. Biophys. Rep..

[22] Wang L., Xu J., Yan Y., Liu H., Karunakaran T., Li F. (2019). Green synthesis of gold nanoparticles from Scutellaria barbata and its anticancer activity in pancreatic cancer cell (PANC-1). Artif. Cells Nanomed. Biotechnol..

[23] Divakaran D., Lakkakula J.R., Thakur M., Kumawat M.K., Srivastava R. (2019). Dragon fruit extract capped gold nanoparticles: Synthesis and their differential cytotoxicity effect on breast cancer cells. Mater. Lett..

[24] Patil M.P., Bayaraa E., Subedi P., Piad L.L.A., Tarte N.H., Kim G. (2019). Do Biogenic synthesis, characterization of gold nanoparticles using Lonicera japonica and their anticancer activity on HeLa cells. J. Drug Deliv. Sci. Technol..

[25] Islam N.U., Jalil K., Shahid M., Rauf A., Muhammad N., Khan A., Shah M.R., Khan M.A. (2019). Green synthesis and biological activities of gold nanoparticles functionalized with Salix alba. Arab. J. Chem..

[26] Jemilugba O.T., Sakho E.H.M., Parani S., Mavumengwana V., Oluwafemi O.S. (2019). Green synthesis of silver nanoparticles using *Combretum erythrophyllum* leaves and its antibacterial activities. Colloids Interface Sci. Commun..

[27] Martini N., Eloff J.N. (1998). The preliminary isolation of several antibacterial compounds from *Combretum erythrophyllum* (Combretaceae). J. Ethnopharmacol..

[28] Martini N.D., Katerere D.R.P., Eloff J.N. (2004). Biological activity of five antibacterial flavonoids from *Combretum erythrophyllum* (Combretaceae). J. Ethnopharmacol..

[29] Bantho S., Naidoo Y., Dewir Y.H. (2020). The secretory scales of *Combretum erythrophyllum* (Combretaceae): Micromorphology, ultrastructure and histochemistry. South Afr. J. Bot..

[30] Mukherjee S., Sau S., Madhuri D., Bollu V.S., Madhusudana K., Sreedhar B., Banerjee R., Patra C.R. (2016). Green synthesis and characterization of monodispersed gold nanoparticles: Toxicity study, delivery of doxorubicin and its bio-distribution in mouse model. J. Biomed. Nanotechnol..

[31] Ovais M., Khalil A.T., Islam N.U., Ahmad I., Ayaz M., Saravanan M., Shinwari Z.K., Mukherjee S. (2018). Role of plant phytochemicals and microbial enzymes in biosynthesis of metallic nanoparticles. Appl. Microbiol. Biotechnol..

[32] Mohamed M.M., Fouad S.A., Elshoky H.A., Mohammed G.M., Salaheldin T.A. (2017). Antibacterial effect of gold nanoparticles against Corynebacterium pseudotuberculosis. Int. J. Vet. Sci. Med..

[33] Gentry S.T., Kendra S.F., Bezpalko M.W. (2011). Ostwald ripening in metallic nanoparticles: Stochastic kinetics. J. Phys. Chem. C.

[34] Dong L., Craig M.M., Khang D., Chen C. (2012). Applications of nanomaterials in biology and medicine. J. Nanotechnol..

[35] Rheder D.T., Guilger M., Bilesky-José N., Germano-Costa T., Pasquoto-Stigliani T., Gallep T.B.B., Grillo R., Carvalho C.D.S., Fraceto L.F., Lima R. (2018). Synthesis of biogenic silver nanoparticles using Althaea officinalis as reducing agent: Evaluation of toxicity and ecotoxicity. Sci. Rep..

[36] Mamonokane O.D., Eunice U.-J., Mahloro H.S.-D. (2018). The antibacterial activity of bacterial endophytes isolated from Combretum molle. Afr. J. Biotechnol..

[37] Ansari M.A., Kalam A., Al-Sehemi A.G., Alomary M.N., AlYahya S., Aziz M.K., Srivastava S., Alghamdi S., Akhtar S., Almalki H.D. (2021). Counteraction of Biofilm Formation and Antimicrobial Potential of Terminalia catappa Functionalized Silver Nanoparticles against Candida albicans and Multidrug-Resistant Gram-Negative and Gram-Positive Bacteria. Antibiotics.

[38] Hu X., Xu X., Fu F., Yang B., Zhang J., Zhang Y., Binte Touhid S.S., Liu L., Dong Y., Liu X. (2020). Synthesis of bimetallic silver-gold nanoparticle composites using a cellulose dope: Tunable nanostructure and its biological activity. Carbohydr. Polym..

[39] Fanoro O.T., Oluwafemi O.S. (2020). Bactericidal antibacterial mechanism of plant synthesized silver, gold and bimetallic nanoparticles. Pharmaceutics.

