# Supplementary material for: Facile Green, Room-Temperature Synthesis of Gold Nanoparticles Using Combretum erythrophyllum Leaf Extract: Antibacterial and Cell Viability Studies against Normal and Cancerous Cells
Source: Antibiotics (Basel). 2021 Jul 22;10(8):893. doi: 10.3390/antibiotics10080893 (PMC8388653; doi:10.3390/antibiotics10080893)
Supplement: Supplementary file 1 [file antibiotics-10-00893-s001.zip › antibiotics-1292627-supplementary.pdf]

Facile Green, Room-Temperature Synthesis of Gold Nanoparticles Using *Combretum Erythrophyllum* Leaf Extract: Antibacterial and Cell Viability Studies against Normal and Cancerous Cells

Supporting Document.

Olufunto T. Fanoro [1,3], Sundararajan Parani [2,3], Rodney Maluleke [2,3], Thabang C. Lebepe [2,3], R. Jose Varghese [2,3] Vuyo Mavumengwana [1] and Oluwatobi S. Oluwafemi [2,3]*

[1] *Department of Biotechnology, University of Johannesburg, Doornfontein, Johannesburg 2028, South Africa*

[2] *Department of Chemical Sciences (formerly applied Chemistry), University of Johannesburg, P.O. Box 17011, Doornfontein, Johannesburg 2028, South Africa*

[3] *Centre for Nanomaterials Sciences Research, University of Johannesburg, Johannesburg, South Africa*

* Corresponding author: oluwafemi.oluwatobi@gmail.com

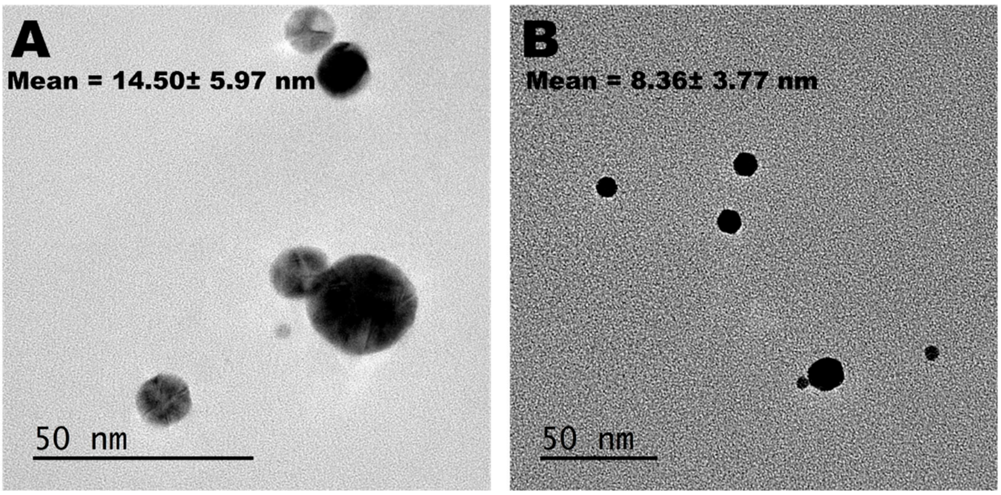

Figure S1. TEM Image of AuNPs synthesized using 0.5 mL of CE (A) at 0 min (B) at 48 h.

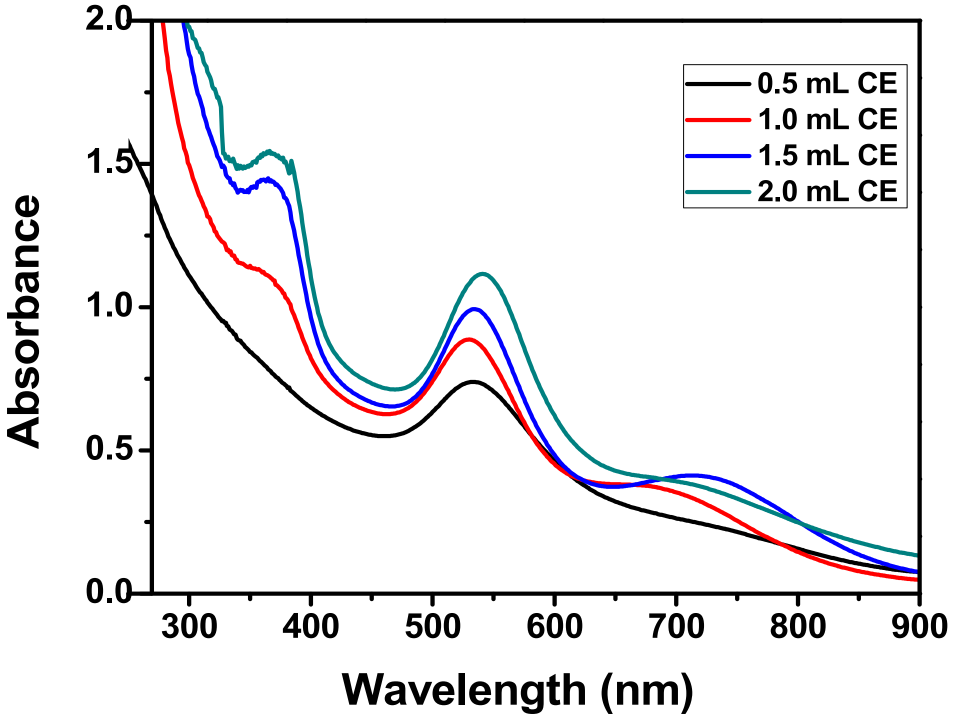
0.5 mL CE
1.0 mL CE
1.5 mL CE
2.0 mL CE
Absorbance
Wavelength (nm)
0.0
0.5
1.0
1.5
2.0
300
400
500
600
700
800
900

(A)

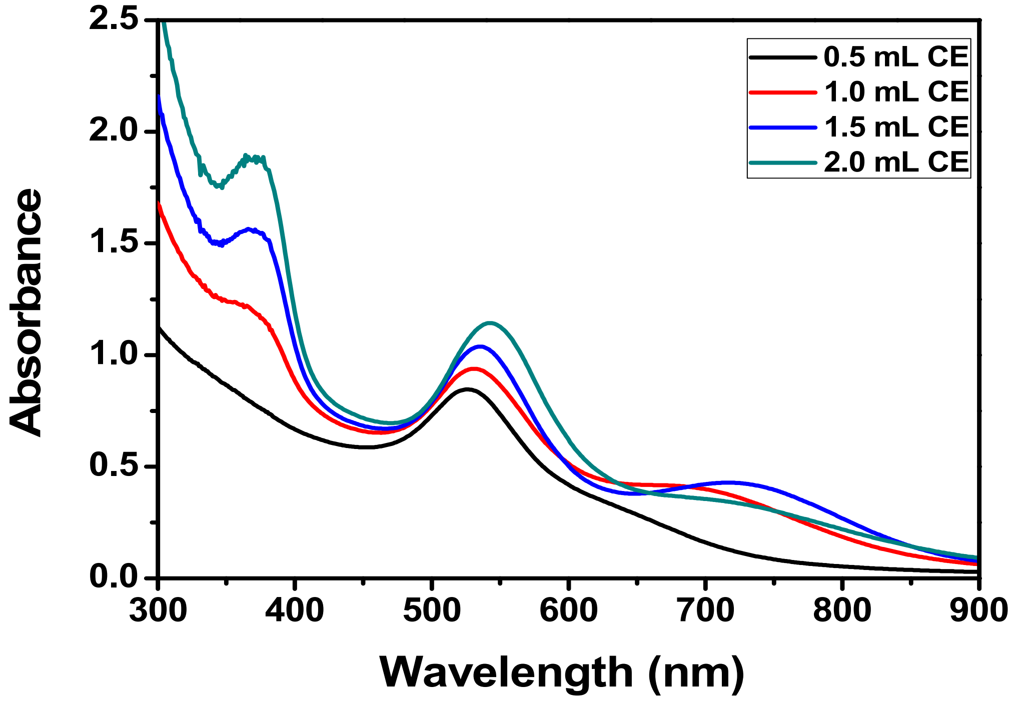
0.5 mL CE
1.0 mL CE
1.5 mL CE
2.0 mL CE
Absorbance
Wavelength (nm)
0.0
0.5
1.0
1.5
2.0
2.5
300
400
500
600
700
800
900

(B)

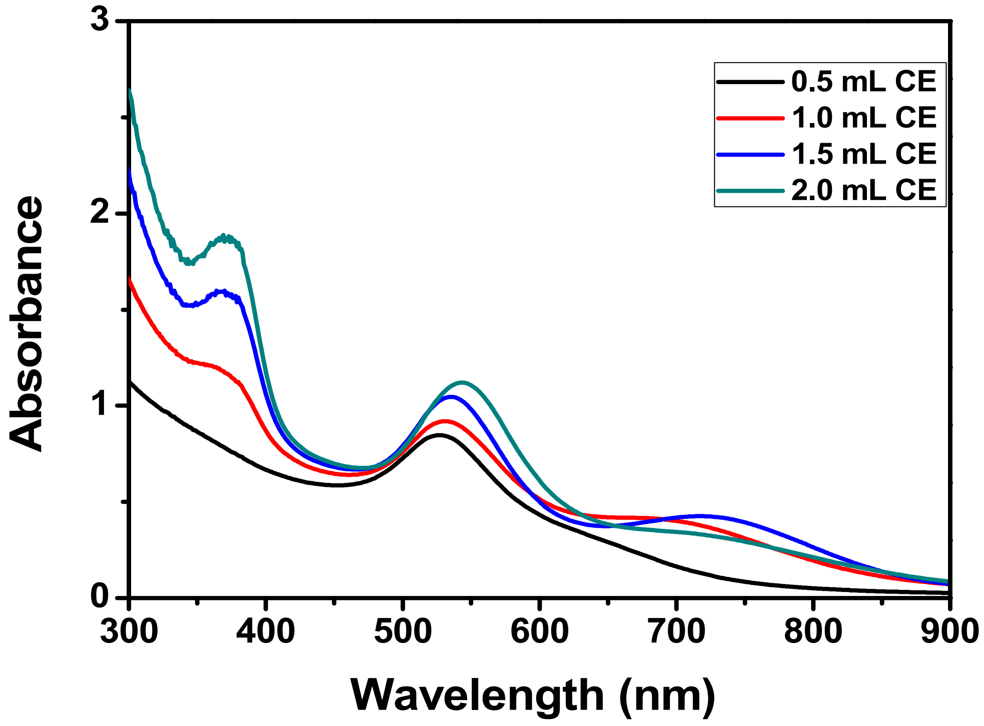

(C)

Figure S2. (**A**). Absorption spectra of AuNPs using Different volume of CE at 0 min. (**B**) Absorbance spectra of AuNPs using different volumes of CE at 24 h. (**C**) Absorbance spectra of AuNPs using different volumes of CE at 48 h.

Table S1. Wavelength and Absorption of the AuNPs using different volumes of CE.

	S/N	0.5 mL CE		1.0 mL CE		1.5 mL CE		2.0 mL CE	
1.		Wave-Length (nm)	Absor-bance	Wave-Length (nm	Absor-bance	Wave-Length (nm	Absor-bance	Wave-Length (nm	Absor-bance
2.	0 min	533	0.74	530	0.89	534	0.99	541	1.12
3.	24h	526	0.85	531	0.94	535	1.0	542	1.14
0	48 h	526	0.85	532	0.92	535	1.05	543	1.12

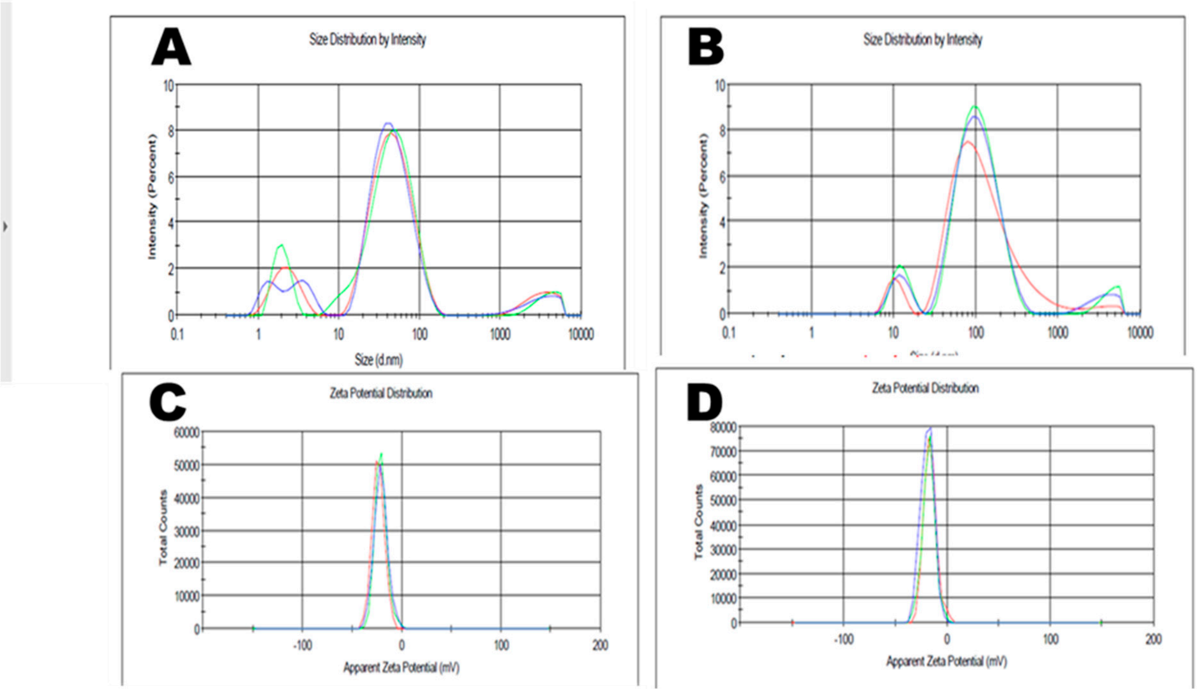

Figure S3. Particle size distribution of AuNPs using (**A**) 1mL CE (B) 2 mL CE, Zeta potential distribution of AuNPs using (**C**) 1 mL CE (**D**) 2 mL CE at 48 h.

Table S2. DLS Analysis of AuNPs AT 48 h.

S/N	Sample	Size (d)nm	Zeta (mV)
1.	AuNPs 1.0 mL CE 48 h	26.39	-18.5
2.	AuNPs 2.0 mL CE 48 h	73.77	-19.2
3.	CE Extract		-15.6

Table S3. Wavelength (nm) of AuNPs using different volumes of CE for 60 Days.

S/N	Sample	Day 0	Day 15	Day 30	Day 45	Day 60
1.	0.5 mL CE	526 nm	535 nm	557 nm	578	591
2.	1.0mL CE	532 nm	532 nm	532 nm	532	533
3.	1.5 mL CE	535 nm	535 nm	535 nm	535	536
4.	2.0 mL CE	543 nm	543 nm	544 nm	544	544

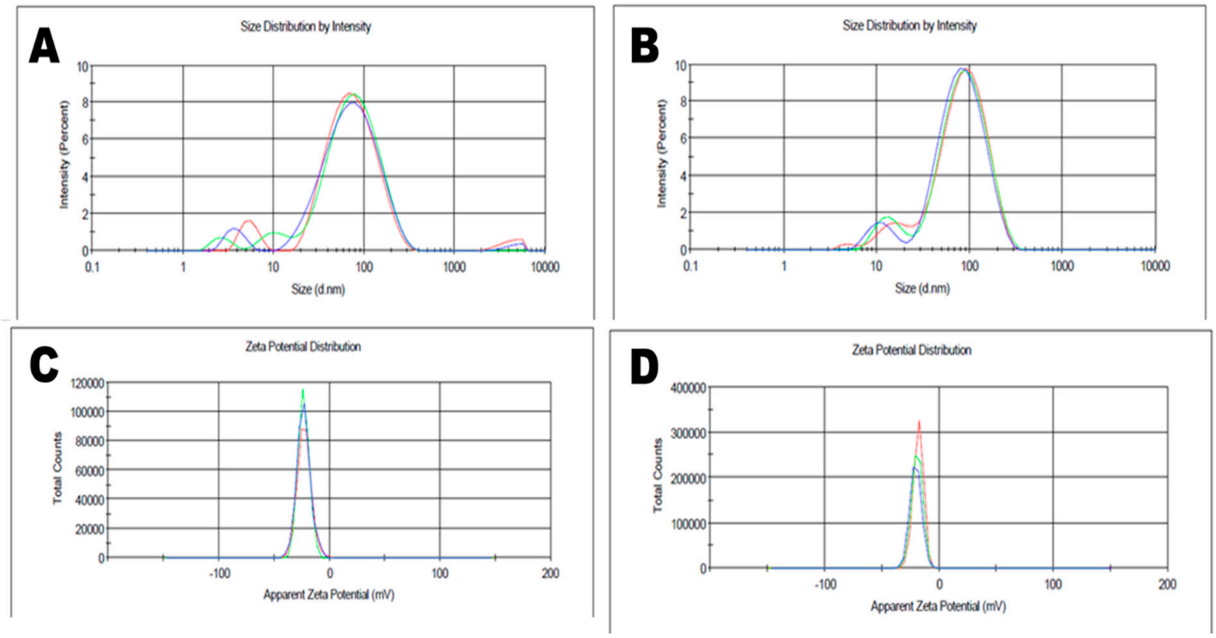

Figure S4. Particle size distribution of AuNPs using (**A**) 1 mL CE (**B**) 2 mL CE, Zeta potential distribution of AuNPs using (**C**) 1 mL CE (**D**) 2 mL CE after 60 days.

Table S4. DLS Analysis of AuNPs after 60 days.

S/N	Sample Name	Size (d. nm)	Zeta (mV)
1	1 mL CE AuNPs	49.76	-23.3
2	2 mL CE AuNPs	58.25	-20.4

Table S5. Antibacterial Activity of AuNPs using 1 mL and 2 mL of CE by MIC.

S/N	Bacterial Strain	Gram +/-	1 mL CE AuNPs (µg/mL)	2 mL CE AuNPs (µg/mL)	CE extract
1	*Staphylococcus epidermidis* (Se) (ATCC14990)	+Ve	62.5	62.5	-
2	*Proteus mirabilis* (Pm) (ATCC 7002)	-Ve	62.5	62.5	-
3	*Escherichia coli* (Ec) (ATCC 25922)	-Ve	62.5	62.5	-
4	*Staphylococcus aureus (Sa)* (ATCC 25923)	+Ve	62.5	62.5	-
5	*Klebsiella pneumoniae (Kp)* (ATCC 13822)	-Ve	62.5	62.5	125
6	*Klebsiella oxytoca* (Ko) (ATCC 8724)	-Ve	62.5	62.5	-
7	*Mycobacterium smegmatis* (Ms) (MC 2155)	+Ve	62.5	62.5	2000
8	*Klebsiella aerogenes* (Ka) (ATCC 27853)	-Ve	2000	2000	-
9	*Enterobacter cloacae* (Ecl) (ATCC 13047)	-Ve	2000	-	-
10	*Proteus vulgaris* (Pv) (ATCC 6380)	-Ve	2000	-	-
11	*Bacillus subtilis* (Bs) (ATCC 19659)	+Ve_	2000	-	2000
12	*Enterococcus faecalis (Ef) (ATCC 13047)*	+Ve	-	-	-
